# Prevalence and distribution pattern of mood swings in Thai adolescents: a school-based survey in the central region of Thailand

**DOI:** 10.1186/s12888-020-02605-0

**Published:** 2020-04-29

**Authors:** Suleemas Angsukiattitavorn, Acharaporn Seeherunwong, Rungnapa Panitrat, Mathuros Tipayamongkholgul

**Affiliations:** 1 D.N.S. Candidate, Faculty of Nursing, Mahidol University, Bangkok, Thailand; 2 Department of Mental Health and Psychiatric Nursing, Faculty of Nursing, Mahidol University, Bangkok, Thailand; 3 Faculty of Nursing HRH Princess Chulabhorn College of Medical Science, Chulabhorn Royal Academy, Bangkok, Thailand; 4 Department of Epidemiology, Faculty of Public Health, Mahidol University, Bangkok, Thailand

**Keywords:** Mood swings, Adolescents, Prevalence, Distribution

## Abstract

**Background:**

Mood swings (MS) are a widely discussed psychiatric ailment of youthful patients. However, there is a lack of research about MS in this population.

**Methods:**

A school-based, cross-sectional study was conducted to investigate the prevalence and distribution pattern of mood swings due to personal and contextual determinants in Thai adolescents in the central region of Thailand. Participants were 2598 students in high schools and vocational schools in Bangkok and three provinces in the central region of Thailand.

**Results:**

The prevalence of mood swings was 26.4%. It was highest among vocational students in Bangkok at 37.1%. MS were more common in adolescents who exhibited risk behaviors and who resided in hazardous situations. The probabilities of MS by characteristic in 15–24 years olds were: bullying involvement 36.9% (*n* = 1293), problematic social media use 55.9%(*n* = 127), high expressed emotion in family 36.6% (*n* = 1256), and studying in a vocational program 29.5% (*n* = 1216) and school located in Bangkok 32.4% (*n* = 561). Also, substance use was a risk for MS with cannabis use at 41.8%(*n* = 55) and heroin use at 48.0% (*n* = 25). Hierarchical logistic regression analysis showed that female gender, having a family history of mental problems, bullying involvement, problematic social media use, high expression of emotion in the family, and the interaction between vocational program enrollments and metropolitan/urban residence associated adolescent mood swings (*p* < .05).

**Conclusions:**

Findings indicate that the pattern of mood swings was associated with significant bullying involvement, social media use, family circumstance, and school characteristics. The public needs greater awareness of MS patterns and the positive implications of MS screening. Early preventive interventions that may limit later mental illness are needed.

## Background

Mental disorders represent a significant public health concern worldwide. Globally, 18–36% of the total population has a mental health problem such as depression, anxiety, and thought disorders [1]. Mental disorders are associated with early mortality and all-cause burden of disease, contributing to 32.4% of years lived with disability (YLDs) and 13% of disability-adjusted life years (DALYs) [2]. Also, these diseases will cost the global economy through lost productivity [3]. Adolescents and young adults are at elevated risk for mental health problems, with 3–22% of school-age children showing symptoms of mental health problems [4] and a majority of these symptoms continuing into adulthood. Identifying premonitory signs of mental disorders in children can lead to early access to treatment and the reduction of negative sequelae of mental illness. Mood swings (MS) represent a common first sign of mental health problems among adolescents who feel stress and are unable to control their moods.

Mood swings refer to abnormal mood changes that are characterized by oscillation, intensity, and inability to regulate affect change, triggered by the environment but without a recognized, activating source. There are a variety of terms referring to these changes, such as mood swings, affective instability, and mood dysregulation, [5–9]. MS has more significantly become associated with general anxiety disorder, major depressive disorder, bipolar disorder and borderline personality disorder [10–12]. Evidence shows that nearly 90% of patients with mental health problems and psychotic symptoms have ten-fold levels of MS when compared with the general population [13]. People with mood swings have twice the risk of suicide as compared to those without MS [14].

In Thailand, a nationwide survey reported an increased rate of mental health problems in adolescents as seen worldwide [15], with a corresponding delay in access to adequate mental health services. MS recognition helps to identify early phases of psychological problems, and further to help reduce the loss of quality of life as well as help alleviate the burden on social resources. MS prevalence can guide policy management and outline the provision and scaling up of appropriate mental service for adolescents. The principal of epidemiology requires an understanding of the distribution of determinants influencing the situation of interest. Generally, critically personal characteristics are associated with mental health problems. Beyond the primary traits of a person, the consequences of exposure to a hazardous situation such as poor communication in the family [16, 17] and a high rate of bullying in school [18, 19] are also crucial for adolescents.

The purposes of this study were to assess the prevalence and distribution patterns of mood swings in Thai adolescents and identify adolescent groups and their associated personal and contextual determinants of mood swings.

## Methods

### Setting

Thailand is a country of Southeast Asia, composed of 76 provinces and comprised of several distinct geographies in four regions, the Northern, Northeastern, Central, and Southern. Bangkok is its capital city with the highest population density; it is a metropolitan province of 22 provinces in the central region. Thai provinces are divided administratively into a central district and other districts. Compulsory education in Thailand has been twelve years since 1997. In grades 10–12, upper secondary education has two tracts: high school and vocational school. The high school track is a program focused on increasing specific knowledge and skills in line with the capacities, aptitudes, and interests of individual learners for academic and technological applications. The vocational education program aims to develop the knowledge and skills of the workforce to match specific job market needs [20]. According to a previous study, high-risk behavior and mental problems amongst Thai youth were highest within areas of the central region and in vocational schools [21]. Thus, this study is a cross-sectional study focused on adolescents of grades 10–12 in high schools and vocational schools in three provinces of the central region of Thailand plus the Bangkok metropolitan area. A stratified, proportional two-cluster sampling approach was used. The first stage of random-cluster sampling included eight schools within each province and three classrooms in each school of the secondary cluster.

The estimated upper secondary students of the central region of Thailand in 2015 was 628,059 [20]. The Lemeshow & Stroh (1988) [22] method was used to determine required the sample size. The proportion of mood instability was based on previous studies at 12–16% [14, 23]. To achieve a precision of ±5% with, a 95% confidence interval, the estimated error was 10%. Based on previous evidence, we assumed a 30% uncompleted questionnaire return rate using a web-based instrument. The total required sample size was calculated to be 2613. The inclusion criteria for students were: 1) 15 years old or above; 2) having a basic grounding in their mother tongue - Thai; 3) having access to modern technology including mobile phones and the internet. A total of 2618 adolescents in 26 schools agreed to participate and 2598 questionnaires were completed (99.24%) and returned via a smartphone application.

### Measures

For mood swings assessment, we used the Affective Lability Scale-Short Form (ALS-SF) modified by Oliver and Simons (2004) [9, 24] which contains 18 items. It used an anchored four-scale (0–3), ranging from “Very uncharacteristic of me” to “Very characteristic of me”. Responses covered three domains: depression/elation (DE, eight items), anxiety/depression (AD, five items) and anger (A, five items). The median score of three domains of ALS-SF was used as a cut-point of MS. Cronbach’s alpha was .91 overall.

*Personal determinants*. Data were provided by sex (male/female/ nonnormative gender), and family history of mental problems divided into three categories (no/yes/uncertain).

The bullying involvement measure was the Illinois Bully Scale (IBS) [25]. IBS contains 18 items with questions scored on a five-level Likert subscale. Cronbach’s alpha was .88 overall.

The Internet Addict Test (IAT) [26] was a self-assessment for social media use. Social media use refers to the use of application services on internet networks, including Chat, Facebook, Instagram, Skype, Twitter and Line via computer, laptop, and mobile phone. The IAT contains 20 items assessed along a six-level scale (0–5). Cronbach’s alpha in this study was .92.

Substance use was measured by the Alcohol, Smoking, and Substance Involvement Screening Test (ASSIST-Lite), Thai version translated by Sawitri Assanangkornchai (2016) [27]. ASSIST-Lite classifies substance into seven categories: alcohol, tobacco, cannabis, opioid, sedatives, stimulants, other unknown. In this study, we added a question of energy drinks as recent evidence reports a high rate of energy drink use among Thai youth [28]. There were three items of the sub-question in each category and yes/ no scoring. The cutoff score associated with “likely substance use disorder” was 2. Cronbach’s alpha in this study was .83.

*Contextual determinants* comprised the family context and school context. The family characteristics indicated family structure (both parents together/ single parent/ father or mother remarried/ foster) and family circumstance refers to expressed emotion in the family. Expressed emotion in the family [29] was assessed using the Level of Expressed Emotion Scale – 46 (LEE-46) as modified by William W. Hale III in 2014. It consisted of 5 domains: Lack of emotional support (LES; 19 items), Intrusiveness (INTR; 7 items), Irritation (IRR; 7 items), Criticism (C; 5 items), and Positive Criticism (PC; 8 items). The answers were scored from anchor-words from untrue to true (1–4). Cronbach’s alpha in this study was .87.

School characteristics measured the education program and the location of the school. The education program was classified into two categories: 1) general school and 2) vocational school. The sites of the schools were categorized: 1) suburban, 2) urban, and 3) Bangkok.

The authors applied the translation processes of the forward and back-translation method specified in WHO guidelines for ALS-SF, IBS, IAT, and LEE-46.

### Statistical analysis

Descriptive statistics were used to describe all study variables. Median was used to describe continuous variables with non-normal distribution. The Kolmogorov-Smirnov test was used for distribution with a significance level of .05, which resulted in 15 outlier cases for further discussion. MS and all determinants were treated as distinct categories for univariate binomial testing. When Chi-square test was statistically significant, Bonferroni Post Hoc test was used to adjust *p*-value for multiple comparisons. Logistic regression was also assessed. The spearman-rank test for bivariate correlation was statistically significant at the .01 level, while not high (r_s_ < .5) or violating the multicollinearity assumption. Five separate hierarchical logistic regression models were employed to identify the association between independent factors and mood swings. The logistic regression statistic was used for data analysis and statistically significant was considered at *p*-value < 0.05. The coefficient of determinants and deviance statistics were considered; − 2 Log likelihoods determined how the fit model for data and values predicted by the model differed from observed values. All selected variables with stay probabilities close to one were included in the model by the enter method. At each step, Akaike’s Information Criterion (AIC) was calculated, and the model with the lowest AIC value chosen as the final model that Hosmer & Lemeshow showed as an acceptable model fit.

## Results

The participants’ mean age was 16.88 years (S.D = 1.10, range 15–24) with 59.6% female and 8.7% of participants reporting a family history of mental problems. Almost one-half of the participants had experienced bullying (49.8%, median 13; Q1 = 8, Q3 = 21). Many participants (41.9%) indicated that they had experienced problematic social media use (Table 1).

**Table 1:** Number and percentage of participants on personal and contextual determinants

Variables	Total Number (%) (*n* = 2598)	Number (%) on Location of school (*n* = 2593^a^)		
		Bangkok (*n* = 561)	Urban (*n* = 1095)	Suburban (*n* = 937)
Sex				
Male	884 (34.0)	292 (52.0)	389 (35.5)	203 (21.7)
Female	1549 (59.6)	238 (42.4)	626 (57.2)	680 (72.6)
Creative gender	165 (6.4)	31 (5.5)	80 (7.3)	54 (5.7)
Age (years)				
15 – 16	1000 (38.5)	198 (35.3)	461 (42.1)	338 (36.1)
17 – 18	1517 (58.4)	354 (63.1)	577 (52.7)	586 (62.5)
≥19	81 (3.1)	9 (1.6)	57 (5.2)	13 (1.4)
Family history of mental problems				
No	1991 (76.6)	418 (74.5)	821 (75.0)	748 (79.8)
Yes	226 (8.7)	53 (9.4)	115 (10.5)	58 (6.1)
- father/mother/sibling	136 (5.2)	34 (6.1)	69 (6.3)	33 (3.5)
- grandparents/uncle/aunt	77 (3.0)	18 (3.2)	37 (3.4)	22 (2.3)
- cousin	13 (0.5)	1 (0.2)	9 (0.8)	3 (0.3)
Uncertain/Unknown	381 (14.7)	90 (16.0)	159 (14.5)	131 (14.0)
Mood Swings (MS)				
Mood swings (AD>5&DE>9&A>5)	686 (26.4)	182 (32.4)	296 (27.0)	206 (22.0)
- Anxiety/Depression (AD) (Above norm=>5)	255 (48.3)	315 (56.1)	516 (47.1)	420 (44.8)
- Depression/Elation (DE) (Above norm=>9)	1210 (46.6)	302 (53.8)	484 (44.2)	422 (38.5)
- Anger (A) (Above norm=>5)	1062 (40.9)	256 (45.6)	455 (41.6)	349 (37.2)
Bullying (IBS)				
Bullying involvement (Above norm=>13)	1293 (49.8)	308 (54.9)	555 (50.7)	427 (45.6)
- Bully behavior (Above norm=>6)	1285 (49.5)	315 (56.1)	542 (49.5)	425 (45.4)
- Fight behavior (Above norm=>3)	1034 (39.8)	242 (43.1)	474 (43.3)	316 (33.7)
- Victim behavior (Above norm=>3)	1279 (49.2)	295 (52.6)	550 (50.2)	431 (46.0)
Social Media Use (IAT)				
- Normal (score < 49)	1383 (53.2)	287 (51.1)	616 (56.3)	479 (51.1)
- Trend to impact (score 50 - 79)	1088 (41.9)	243 (43.3)	431 (39.4)	410 (43.8)
- Get impact/problems (score > 80)	127 (4.9)	31 (5.5)	48 (4.4)	48 (5.1)
1^st^ order reason to use social media				
- Find someone to chat	625 (24.1)	132 (23.5)	260 (23.7)	232 (24.8)
- Play game	665 (25.6)	137 (24.4)	276 (25.2)	250 (26.7)
- Communicate in family	552 (21.2)	123 (21.9)	212 (19.3)	216 (23.1)
- Entertainment	828 (31.9)	182 (32.4)	322 (29.4)	323 (34.5)
- Chat with boy/girlfriend	732 (28.2)	173 (30.8)	291 (26.6)	268 (28.6)
- Education/knowledge	390 (15.0)	84 (15.0)	164 (15.0)	142 (15.2)
Substance use in 3 months past				
- Tobacco (score>2)	61 (2.3)	12 (2.1)	43 (3.9)	6 (0.6)
- Alcohol (score>3)	387 (14.9)	76 (13.5)	169 (15.4)	142 (15.2)
- Cannabis (score>2)	55 (2.1)	8 (1.4)	32 (2.9)	15 (1.6)
- Stimulant (score>2)	33 (1.3)	4 (0.7)	18 (1.6)	11 (1.2)
- Sedative (score>2)	45 (1.7)	7 (1.2)	26 (2.4)	12 (1.3)
- Heroin (score>2)	25 (1.0)	3 (0.5)	13 (1.2)	9 (1.0)
- Other substance^b^	63 (2.4)	11 (2.0)	32 (2.9)	20 (2.1)
- Energy drinking (score>2)	351 (13.5)	89 (15.9)	150 (13.7)	112 (12.0)
Family structure				
- Both parents are together	1632 (62.8)	370 (66.0)	653 (59.6)	604 (64.5)
- Single parent	847 (32.6)	160 (28.5)	386 (35.3)	301 (32.1)
- Father/mother remarried	95 (3.7)	22 (3.9)	44 (4.0)	29 (3.1)
- Foster	24 (0.9)	9 (1.6)	12 (1.1)	3 (0.3)
Family circumstance:				
Expressed emotion in family (EE)				
High EE (Above norm= > 108)	1256 (48.3)	307 (54.7)	536 (48.9)	412 (44.0)
- Lack of support (LES) High LES (Above norm=>40)	1185 (45.6)	300 (53.5)	510 (46.6)	375 (40.0)
- Intrusive (INTR) High INTR (Above norm=>18)	1295 (49.2)	266 (47.4)	575 (52.5)	451 (48.1)
- Irritation (IRR) High IRR (Above norm=>15)	1205 (46.4)	280 (49.9)	507 (46.3)	417 (44.5)
- Criticism (C) High C (Above norm=>11)	1224 (47.1)	283 (50.4)	547 (49.9)	393 (41.9)
- Positive Criticism (PC) High PC (Above norm=>24)	987 (38.0)	195 (34.8)	406 (37.1)	381 (40.6)
Educational program				
- High school	1382 (53.2)	227 (40.5)	489 (44.7)	662 (70.7)
- Vocational school	1216 (46.8)	334 (59.5)	606 (55.3)	275 (29.3)

^a^five were missing data of school variables

^b^Other substance use groups, participated were reported, identified Pro (procodyl), B5 (benzhexol), tramadol, codeine

### Prevalence and distributions of mood swings

**Fig. 1:**
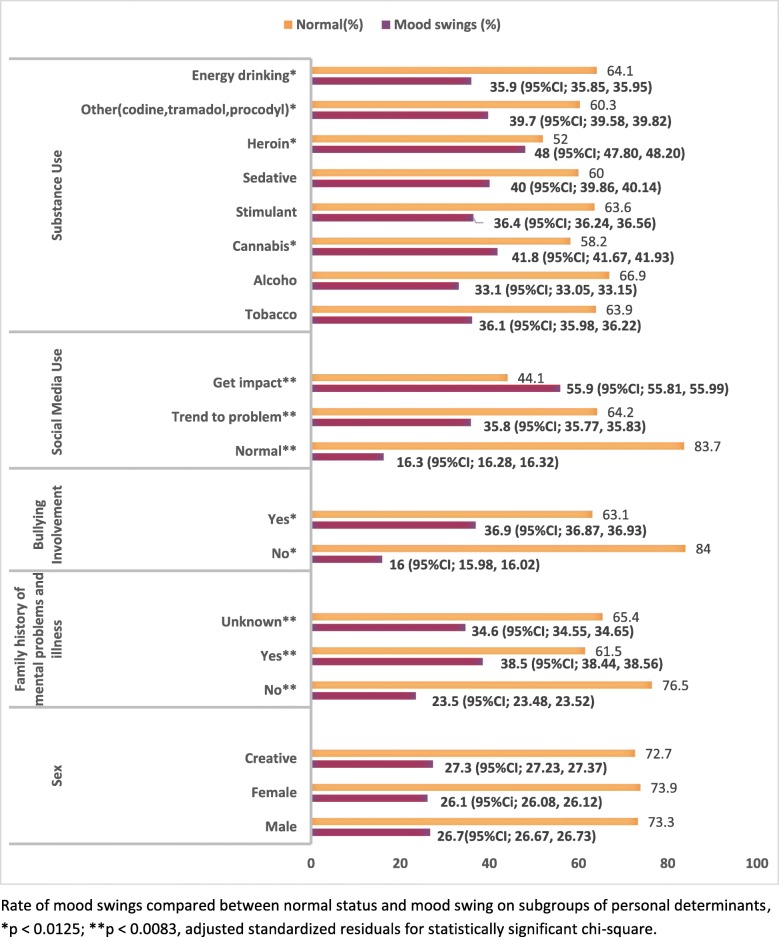
Rate of mood swings compared between normal status and mood swing on subgroups of personal determinants, *p < 0.0125; **p < 0.0083, adjusted standardized residuals for statistically significant chi-square

**Fig. 2:**
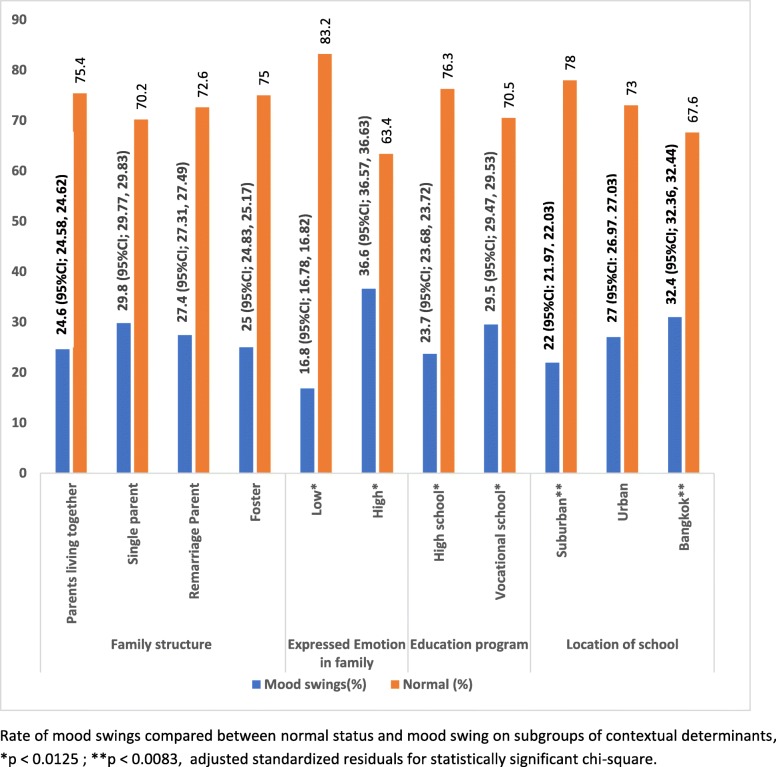
Rate of mood swings compared between normal status and mood swing on subgroups of contextual determinants, *p < 0.0125; **p < 0.0083, adjusted standardized residuals for statistically significant chi-square

The prevalence of mood swings was 26.4%. We compared distribution of MS within present and absent of determinant in each subgroup of personal and contextual presented in Figs. 1 and 2. According to the personal determinants, the participants who had problematic social media use had the highest prevalence rate of mood swings (55.9%).

Regarding the contextual determinants (Fig. 2.), the probability of the participants who were living in high expressed emotion in the family was a risk to mood swings, 36.6%. The vocational school was probably risk to mood swings more than high school (29.5% vs 23.7%). Location of the school, Bangkok was a risk to mood swings more than urban and suburban areas.

### Potential of personal and contextual determinants associated with mood swings

In all, 2598 students have completed the ALS-SF questionnaire for mood swings screening. However, five were missing data of school variables so 2593 students were included for model analysis. Evaluation of logistic regression models includes the assessment of multicollinearity and outliers. Exclusion of the outlier 15 cases did not improve the performance of the model, nor changed the significance of the parameter estimates for variables in the equation. Thus, the outlier cases were retained, and the original model was further evaluated and interpreted.

The analysis showed that there was a difference between -2Log likelihood of a null or initial model and a full model (−2Log likelihood changed = 361.64). Also, the Hosmer & Lemeshow Statistic test is 398.53 (df = 20, *p* = .106). These two indexes demonstrated that the full model is fitted.

Significant OR produced by the hierarchical logistic regression analyses are displayed in Table 2. The participants who had the family history of mental problems were twice as likely to have MS as those without (OR_adj_ = 2.04; CI 1.53, 2.72; *p* < .001) in the Model I. After controlling for unmodifiable person variables in Model II, those with bullying involvement were twice as likely to have MS as those with low/ never bullying (OR_adj_ = 2.381; CI 1.952, 2.904; *p* < .001). Problematic social media use produced the highest MS risk, five times that of the standard group (OR_adj_ = 5.080; CI 3.425, 7.533; *p* < .001). Those with a tendency for such use were twice as likely to have MS as the standard group (OR_adj_ = 2.394; CI 1.963, 2.920; *p* < .001). Because some substance use sub-groups were too small, we grouped substance use into four categories (tobacco/alcohol drinking/ energy drinking/ illicit substance), with only illegal substance use (cannabis, stimulant, sedative, heroin and other substances such as tramadol, codeine, and procodyl) in the past three months related to MS (OR_adj_ = 1.751; CI 1.103, 2.780; *p* < .05).

**Table 2:** Binary logistic regression analyses of associations between person and contextual determinants and mood swings among Thai adolescent in central region of Thailand (*n* = 2593)

Variable	Model 1			Model 2			Model 3			Model 4			Model 5		
	SE	*P*-value	OR	SE	*P*-value	OR	SE	*P*-value	OR	SE	*P*-value	OR	SE	*P*-value	OR
Constant	.082	.000	.307	.121	.000	.104	.140	.000	.067	.167	.000	.047	.171	.000	.053
**Personal level**															
*Sex*															
Male ^Ref^															
Female	.097	.971	.996	.104	.435	1.085	.106	.122	1.179	.110	.017	1.299	.111	.011	1.325
Creative gender	.192	.957	1.011	.204	.873	.968	.207	.923	1.020	.209	.740	1.072	.210	.627	1.107
*`Family history of mental problems and illness*															
No ^ref^															
Yes	.147	.000	2.042	.157	.000	2.062	.159	.000	1.985	.160	.000	1.969	.161	.000	1.959
Uncertain	.120	.000	1.716	.127	.000	1.588	.130	.002	1.495	.130	.002	1.500	.131	.003	1.481
*Bullying Involvement* (base=below median)	-2LL = 2955.34 χ ^2^ =36.88, df= 4, p <.001 Classification accuracy =73.6 AIC = 2959.34			.101	.000	2.381	.103	.000	2.273	.103	.000	2.267	.103	.000	2.281
*Social media use*															
Normal ^Ref^															
Trend to impact				.101	.000	2.394	.103	.000	2.240	.104	.000	2.272	.104	.000	2.251
Get impact/problems				.201	.000	5.080	.205	.000	4.158	.206	.000	4.174	.206	.000	4.081
*Substance use in past 3 months (base=No)*															
Tobacco				.310	.980	1.008	.310	.803	.926	.314	.624	.857	.315	.511	.813
Alcohol				.136	.772	.961	.138	.513	.914	.140	.455	.901	.140	.504	.911
Energy drinking				.135	.210	1.185	.138	.209	1.189	.138	.206	1.191	.138	.224	1.183
Illicit substance				.236	.017	1.751	.239	.052	1.593	.240	.045	1.618	.242	.054	1.592
**Contextual level**															
*Family structure*				-2LL = 2689.78 χ ^2^ =302.44, df= 11, p <.001 Classification accuracy =75.3 AIC = 2705.78											
Parents are together ^Ref^															
Single parent							.103	.122	1.172	.103	.126	1.171	.104	.146	1.162
Father/mother remarried							.260	.895	1.035	.260	.981	1.006	.261	.945	1.018
Foster							.501	.466	.694	.507	.333	.612	.509	.305	.594
Family circumstance: Expressed emotion in family (Ref=below median)							.100	.000	2.228	.101	.000	2.175	.101	.000	2.178
*Education Program*							-2LL = 2620.01 χ ^2^ =372.21, df= 15, p <.001 Classification accuracy =75.5 AIC = 2640.01								
High school ^Ref^															
Vocational school										.102	.053	1.219	.194	.231	.793
*Location of school*															
Sub-urban ^Ref^															
Urban										.118	.058	1.250	.153	.856	1.028
Bangkok										.138	.001	1.566	.195	.396	1.180
Location of school* Education Program										-2LL = 2601.24 χ ^2^ =390.98, df= 18, p <.001 Classification accuracy =77 AIC = 2625.24					
Sub-urban*High school ^Ref^															
Urban* Vocational school													.247	.023	1.756
Bangkok* Vocational sc.													.282	.013	2.009
													-2LL = 2593.7 χ ^2^ =398.53, df= 20, p <.001 Classification accuracy =76.7 AIC = 2619.7		

The context included family and school characteristics. Only high expressed emotion in the family is associated with mood swings when controlling the person variables (OR_adj_ = 2.228; CI 1.832, 2.710; *p* < .001) as shown in Model III. School characteristics (Model VI), show a tendency for vocational programs to be associated with MS (OR_adj_ = 1.219; CI 0.998, 1.490; *p* = .053) when compared to high school. Urban schools located in Bangkok area were 1.5 times more likely to show MS as sub-urban schools (OR_adj_ = 1.566; CI 1.195, 2.053; *p* < .001), so schools located in urban areas tended to have significantly higher MS (OR_adj_ = 1.250; CI 0.993, 1.575; *p* = .058). The final model, with an interaction between the education program and location of the school showed MS associated with vocational schools in urban and Bangkok areas. The interaction variables produced almost two times the likelihood of mood swings as compared to high schools in sub-urban area (OR_adj_ = 1.756; CI 1.081, 2.851; *p* < .05), (OR_adj_ = 2.009; CI 1.155, 3.494; *p* < .05) respectively. However, results in the whole model of independent variables, Nagelkerke’s pseudo- R-square explained 20.8% of the variance for mood swings.

## Discussion

The study found the prevalence of MS among Thai adolescents was 26.4%. It showed a higher rate of the prevalence compared to previous studies [14, 23], which could be due to the different populations, and sources of data. The population used in this study was adolescent 15–24 years in school-based sample, unlike earlier studies, participants were diagnosed or met criteria for diagnosis with mental problem and illness [23]. Regarding source data in previous studies, it was extracted from a part of the Structured Clinical Interview for DSM-IV Axis II Personality Disorders (SCID-II) [14] and free text extracts clinical information that relevant keywords such as instability, mood, affect and emotion [23]. In the current study, we directly collected data from the participants by using the ALS-18 questionnaire, which included depression, anxiety, elation, and anger, thereby screening several symptoms.

Being female increased the odds of MS supporting previous researches that revealed emotional problems were more common in school-age girls, and by early adulthood, women are more likely to be diagnosed with a mental health condition than men [30, 31]. Attribution explained gender differences in social inequalities, socioeconomic status, ethnicity, sexual orientation, and other factors intersect to impact women throughout the life course affecting their mental health [30].

Bivariate analysis showed the highest MS rate in those of nonnormative gender. Although Thai sex norms are modern formations that have undergone a tremendous transformation over the last century, nonnormative gender continues to be less accepted as a gender norm and is still stigmatized [32]. For this reason, this gender is a stressor.

The family history of mental problems increased the odds of MS as scientific research of the phenotypes studies shown the mental illness in family history supports significant for mental health problems and disorders among relatives [9].

Bullying involvement appeared significantly associated with increased odds of MS is supported by previous work of bully and mental problems [33, 34]. For problematic social media use, the current study shows both increased impact and that problems arising from social media in daily life greatly increased the risk of MS as with psycho-pathologies such as depression, anxiety, and suicidal ideation [35]. The primary developmental crisis in the adolescent period is self-identity (Erikson’s Psychosocial development), the disjunction between a physical change and socially allowed independence. This period, when youth disengage from parents, can result in high levels of conflict and a concurrent status viewed as stressful. It may lead to taking risky behavior.

High expressed emotion in the family increased odds of MS. Family is an essential context for human development and mental health. Interaction patterns, communications, and emotional interactions between family members are associated with anxiety and depression among adolescents [30, 36, 37].

Another study conducted in Thailand showed that health risk behaviors were high among vocational students [20], suggesting that identification and planning of extra activities for delinquent vocational students are required [38] to counter the social stressors of these programs. We found that the prevalence of MS in vocational schools was higher than in general high schools (29.5% vs 23.7%). However, this alone was not a significant effect on MS in the logistic regression model, but the interaction between vocational school and urban and metropolitan locations significantly increased the risk of mood swings as compared to high school programs in the suburbs.

That the location of the school in the metropolitan area increased the odds of MS is supported by previous studies in Western and Asian countries where higher odds of mental problems were found in metropolitan and semi-rural areas than rural areas [39]. The reality of health problems and mental health problems in urban areas may be related to harmful social fragmentation and having low social capital-behavior networks among people [39]. The current study showed that schools in Bangkok had the highest frequency of MS. The final model presented significant increases in risk for mood swings as an interaction of vocational schooling and Bangkok/ urban area, consistent with previous health and mental health studies in Thailand [40]. Although the urban areas of Thailand provide better access to health services, they also expose one to health risk and higher living costs. Many rural areas now have more established medical facilities, and local social norms in rural areas are more supportive that positive health factors than in urban life [41].

Our results differed from previous research which reported significant associations between substance use and mental problems and behavior problems, and suicide risk [42]. With limited numbers, less than 5% of our sample with substance use in subgroups, we did find a positive tendency of illicit substance use such as heroin, sedative use with increased odds of MS.

## Conclusions

Our study contributes to scientific knowledge by providing the first epidemiological data on the prevalence of mood swings among adolescents in the school system. The result indicates a high prevalence of mood swings in total adolescents aged above 15 years at 26.4%. The prevalence is highlight among vocational students in Bangkok at 37.1%. Considering adolescents who exhibited risk behavior, the highest mood swings was found in those who had problematic social media use at 55.9%. Public awareness regarding mood swings should include in mental health policy and early intervention and prevention of mental health problems is provision. Mental health care personnel in the school should have knowledge and skill for providing screening, conducting early interventions, and monitoring and referral to higher-competence professionals, as needed. It is essential to establish and develop the robust contextual support of adolescents. Critical responses to children and adolescent’s emotional and mental health needs from parents and school providers are needed, especially critical in cities.

### Strengths and limitations

The prevalence and distributions of mood swings among school-age adolescents of different sexes, risk-taking behaviors, family circumstances, and residential locations were examined. Also, the knowledge of the personal and contextual determinants of mood swings could help fill the gap in knowledge of mental health in adolescents. The study provides empirical support for MS screening to prevent mental health problems.

As with any study, a significant limitation of the present study is the cross-sectional study design with a natural weakness of the time order necessary for a causal relationship. The present study recruited only youth in the regular school system. Thus, the pattern of mood swings might not be representative of youth outside the educational setting, with youth who have dropped out of the education system.

Based on findings from this and other studies, efforts should be made to more adequately investigate factors that contribute the mental health literacy, including mood management in the school. Longitudinal cohort studies are vital if we are to understand the impact of mood swing on the physiological, behavioral, psychological, social and academic performance of adolescents and manage it.

## Supplementary information

**Additional file 1: Table S1.** Number and percentage of participants on personal and contextual determinants.

**Additional file 2: Table S2.** Binary logistic regression analyses of associations between personal and contextual determinants and mood swings among Thai adolescents.

## Data Availability

The datasets used and analyzed during the current study are available from the School of Graduate, Mahidol University on reasonable request.

## Abbreviations

A: Anger
AD: Anxiety/depression
ALS-SF: Affective Lability Scale-Short Form
ASSIST-Lite: Alcohol, Smoking, and Substance Involvement Screening Test
C: Criticism
DALYs: Disability-Adjusted Life Years
DE: Depression/Elation
IAT: Internet Addict Test
IBS: Illinois Bully Scale
INTR: Intrusiveness
IRR: Irritation
LEE-46: Level of Expressed Emotion scale – 46
LES: Lack of emotional support
MS: Mood Swings
PC: Positive Criticism
YLDs: Years Lived with Disability
