# Supplementary material for: Prevalence and distribution pattern of mood swings in Thai adolescents: a school-based survey in the central region of Thailand
Source: BMC Psychiatry. 2020 Apr 29;20:191. doi: 10.1186/s12888-020-02605-0 (PMC7189499; doi:10.1186/s12888-020-02605-0)
Supplement: Supplementary file 1 — Additional file 1: Table S1. Number and percentage of participants on personal and contextual determinants. [file 12888_2020_2605_MOESM1_ESM.pdf]

Table 1 Number and percentage of participants on personal and contextual determinants

Variables	Total Number (%) (n=2598)	Number (%) on Location of school (n=2593*)		
		Bangkok (n=561)	Urban (n=1095)	Suburban (n=937)
Sex				
Male	884(34.0)	292(52.0)	389(35.5)	203(21.7)
Female	1549(59.6)	238(42.4)	626(57.2)	680(72.6)
Creative gender	165(6.4)	31(5.5)	80(7.3)	54(5.7)
Age (years)				
15 – 16	1000(38.5)	198(35.3)	461(42.1)	338(36.1)
17 – 18	1517(58.4)	354(63.1)	577(52.7)	586(62.5)
≥19	81(3.1)	9(1.6)	57(5.2)	13(1.4)
Family history of mental problems				
No	1991(76.6)	418(74.5)	821(75.0)	748(79.8)
Yes	226(8.7)	53(9.4)	115(10.5)	58(6.1)
- father/mother/sibling	136(5.2)	34(6.1)	69(6.3)	33(3.5)
- grandparents/uncle/aunt	77(3.0)	18(3.2)	37(3.4)	22(2.3)
- cousin	13(0.5)	1(0.2)	9(0.8)	3(0.3)
Uncertain/Unknown	381(14.7)	90(16.0)	159(14.5)	131(14.0)
Mood Swings (MS)				
Mood swings (AD>5&DE>9&A>5)	686 (26.4)	182(32.4)	296(27.0)	206(22.0)
- Anxiety/Depression (AD) (Above norm=>5)	255(48.3)	315(56.1)	516(47.1)	420(44.8)
- Depression/Elation (DE) (Above norm=>9)	1210 (46.6)	302(53.8)	484(44.2)	422(38.5)
- Anger (A) (Above norm=>5)	1062(40.9)	256(45.6)	455(41.6)	349(37.2)
Bullying (IBS)				
Bullying involvement (Above norm=>13)	1293(49.8)	308(54.9)	555(50.7)	427(45.6)
- Bully behavior (Above norm=>6)	1285(49.5)	315(56.1)	542(49.5)	425(45.4)
- Fight behavior (Above norm=>3)	1034(39.8)	242(43.1)	474(43.3)	316(33.7)
- Victim behavior (Above norm=>3)	1279(49.2)	295(52.6)	550(50.2)	431(46.0)
Social Media Use (IAT)				
- Normal (score < 49)	1383(53.2)	287(51.1)	616(56.3)	479(51.1)
- Trend to impact (score 50 - 79)	1088(41.9)	243(43.3)	431(39.4)	410(43.8)
- Get impact/problems (score > 80)	127(4.9)	31(5.5)	48(4.4)	48(5.1)

Table 1 Number and percentage of participants on personal and contextual determinants (cont.)

Variables	Total Number (%) (n=2598)	Number (%) on Location of school (n=2593*)		
		Bangkok (n=561)	Urban (n=1095)	Suburban (n=937)
1st order reason to use social media				
- Find someone to chat	625 (24.1)	132(23.5)	260(23.7)	232(24.8)
- Play game	665 (25.6)	137(24.4)	276(25.2)	250(26.7)
- Communicate in family	552 (21.2)	123(21.9)	212(19.3)	216(23.1)
- Entertainment	828 (31.9)	182(32.4)	322(29.4)	323(34.5)
- Chat with boy/girlfriend	732 (28.2)	173(30.8)	291(26.6)	268(28.6)
- Education/knowledge	390 (15.0)	84(15.0)	164(15.0)	142(15.2)
Substance use in 3 months past				
- Tobacco (score>2)	61 (2.3)	12(2.1)	43(3.9)	6(0.6)
- Alcohol (score>3)	387 (14.9)	76(13.5)	169(15.4)	142(15.2)
- Cannabis (score>2)	55 (2.1)	8(1.4)	32(2.9)	15(1.6)
- Stimulant (score>2)	33 (1.3)	4(0.7)	18(1.6)	11(1.2)
- Sedative (score>2)	45 (1.7)	7(1.2)	26(2.4)	12(1.3)
- Heroin (score>2)	25 (1.0)	3(0.5)	13(1.2)	9(1.0)
- Other substance**	63 (2.4)	11(2.0)	32(2.9)	20(2.1)
- Energy drinking (score>2)	351 (13.5)	89(15.9)	150(13.7)	112(12.0)
Family structure				
- Both parents are together	1632(62.8)	370(66.0)	653(59.6)	604(64.5)
- Single parent	847(32.6)	160(28.5)	386(35.3)	301(32.1)
- Father/mother remarried	95(3.7)	22(3.9)	44(4.0)	29(3.1)
- Foster	24(0.9)	9(1.6)	12(1.1)	3(0.3)
Family circumstance:				
Expressed emotion in family (EE)				
High EE (Above norm= > 108)	1256 (48.3)	307(54.7)	536(48.9)	412(44.0)
- Lack of support (LES)				
High LES (Above norm=>40)	1185 (45.6)	300(53.5)	510(46.6)	375(40.0)
- Intrusive (INTR)				
High INTR (Above norm=>18)	1295 (49.2)	266(47.4)	575(52.5)	451(48.1)
- Irritation (IRR)				
High IRR (Above norm=>15)	1205 (46.4)	280(49.9)	507(46.3)	417(44.5)
- Criticism (C)				
High C (Above norm=>11)	1224 (47.1)	283(50.4)	547(49.9)	393(41.9)
- Positive Criticism (PC)				
High PC (Above norm=>24)	987 (38.0)	195(34.8)	406(37.1)	381(40.6)
Educational program				
- High school	1382(53.2)	227(40.5)	489(44.7)	662(70.7)
- Vocational school	1216(46.8)	334(59.5)	606(55.3)	275(29.3)

* five were missing data of school variables

**Other substance use groups, participated were reported, identified Pro (procodyl), B5 (benzhexol), tramadol, codeine.
