# Supplementary material for: Prevalence and distribution pattern of mood swings in Thai adolescents: a school-based survey in the central region of Thailand
Source: BMC Psychiatry. 2020 Apr 29;20:191. doi: 10.1186/s12888-020-02605-0 (PMC7189499; doi:10.1186/s12888-020-02605-0)
Supplement: Supplementary file 2 — Additional file 2: Table S2. Binary logistic regression analyses of associations between personal and contextual determinants and mood swings among Thai adolescents. [file 12888_2020_2605_MOESM2_ESM.pdf]

Table 2 Binary logistic regression analyses of associations between person and contextual determinants and mood swings among Thai adolescent in central region of Thailand (n = 2593)

Variable	Model 1			Model 2			Model 3			Model 4			Model 5		
	SE	P-value	OR	SE	P-value	OR	SE	P-value	OR	SE	P-value	OR	SE	P-value	OR
Constant	.082	.000	.307	.121	.000	.104	.140	.000	.067	.167	.000	.047	.171	.000	.053
**Personal level**															
Sex															
Male [Ref]															
Female	.097	.971	.996	.104	.435	1.085	.106	.122	1.179	.110	.017	1.299	.111	.011	1.325
Creative gender	.192	.957	1.011	.204	.873	.968	.207	.923	1.020	.209	.740	1.072	.210	.627	1.107
Family history of mental problems and illness															
No [ref]															
Yes	.147	.000	2.042	.157	.000	2.062	.159	.000	1.985	.160	.000	1.969	.161	.000	1.959
Uncertain	.120	.000	1.716	.127	.000	1.588	.130	.002	1.495	.130	.002	1.500	.131	.003	1.481
Bullying Involvement	-2LL = 2955.34														
(base=below median)	χ^2 =36.88, df= 4,			.101	.000	2.381	.103	.000	2.273	.103	.000	2.267	.103	.000	2.281
	p <.001														
Social media use	Classification accuracy														
Normal [Ref]	=73.6														
Trend to impact	AIC = 2959.34			.101	.000	2.394	.103	.000	2.240	.104	.000	2.272	.104	.000	2.251
Get impact/problems				.201	.000	5.080	.205	.000	4.158	.206	.000	4.174	.206	.000	4.081
Substance use in past 3 months (base=No)															
Tobacco				.310	.980	1.008	.310	.803	.926	.314	.624	.857	.315	.511	.813
Alcohol				.136	.772	.961	.138	.513	.914	.140	.455	.901	.140	.504	.911
Energy drinking				.135	.210	1.185	.138	.209	1.189	.138	.206	1.191	.138	.224	1.183
Illicit substance				.236	.017	1.751	.239	.052	1.593	.240	.045	1.618	.242	.054	1.592

Table 2 Binary logistic regression analyses of associations between person and contextual determinants and mood swings among Thai adolescent in central region of Thailand (cont.) (n = 2593)

Variable	Model 1			Model 2			Model 3			Model 4			Model 5		
	SE	P-value	OR	SE	P-value	OR	SE	P-value	OR	SE	P-value	OR	SE	P-value	OR
**Contextual level**				-2LL = 2689.78											
Family structure				χ^2 =302.44, df= 11,											
Parents are together [Ref]				p <.001			.103	.122	1.172	.103	.126	1.171	.104	.146	1.162
Single parent				Classification accuracy			.260	.895	1.035	.260	.981	1.006	.261	.945	1.018
Father/mother remarried				=75.3			.501	.466	.694	.507	.333	.612	.509	.305	.594
Foster				AIC = 2705.78											
Family circumstance: Expressed emotion in family (Ref=below median)							.100	.000	2.228	.101	.000	2.175	.101	.000	2.178
Education Program							-2LL = 2620.01								
High school [Ref]							χ^2 =372.21, df= 15,								
Vocational school							p <.001			.102	.053	1.219	.194	.231	.793
Location of school							Classification accuracy								
Sub-urban [Ref]							=75.5								
Urban							AIC = 2640.01			.118	.058	1.250	.153	.856	1.028
Bangkok										.138	.001	1.566	.195	.396	1.180
Location of school Education Program*										-2LL = 2601.24					
										χ^2 =390.98, df= 18,					
Sub-urban*High school [Ref]										p <.001					
Urban* Vocational school										Classification accuracy			.247	.023	1.756
Bangkok* Vocational sc.										=77			.282	.013	2.009
										AIC = 2625.24			-2LL = 2593.7		
													χ^2 =398.53, df= 20,		
													p <.001		
													Classification accuracy		
													=76.7		
													AIC = 2619.7		
